# Prevention Strategies for All Hospital-Onset Urinary Tract Infections: Best Practice Consensus Recommendations

**DOI:** 10.1093/ofid/ofag060

**Published:** 2026-02-06

**Authors:** Edward J Septimus, Lily A Arya, Rebecca Crapanzano-Sigafoos, Roger Dmochowski, Opal Dy, JoAnn Emer-Seltun, Sandra Engberg, Robert Garcia, Mikel Gray, Michael Kennelly, Sarah Krein, Jennifer Meddings, Rekha Murthy, Diane K Newman, AnnMarie Pettis, Sara Reese, Emily Sidlow, Kathleen Vollman

**Affiliations:** Department of Population Medicine, Harvard Medical School and Harvard Pilgrim Health Care Institute, Boston, Massachusetts, USA; Department of Obstetrics and Gynecology, Division of Urogynecology and Pelvic Reconstructive Surgery, Perelman School of Medicine, University of Pennsylvania, Philadelphia, Pennsylvania, USA; Center for Research, Practice and Innovation, Association for Professionals in Infection Control and Epidemiology (APIC), Arlington, Virginia, USA; Department of Urology, Vanderbilt University Medical Center, Nashville, Tennessee, USA; Department of Nursing, Arrowhead Regional Medical Center, Colton, California, USA; Educator WEB WOC® Programs, Minneapolis, Minnesota, USA; MercyOne North Iowa Continence Clinic, Vascular Wound Center, Mason City, Iowa, USA; University of Pittsburgh School of Nursing, Pittsburgh, Pennsylvania, USA; Independent Consultant in Infection Prevention, Enhanced Epidemiology LLC, NewYork, New York, USA; Department of Urology, University of Virginia, Charlottesville, Virginia, USA; Atrium Health Women's Care Urogynecology & Pelvic Surgery, Charlotte, North Carolina, USA; Department of Internal Medicine, University of Michigan North Campus Research Complex (NCRC), Ann Arbor, Michigan, USA; Department of Internal Medicine, University of Michigan Medical School, Ann Arbor, Michigan, USA; Center for Clinical Management Research, Veterans Affairs Ann Arbor Healthcare System, Ann Arbor, Michigan, USA; Department of Pediatrics, University of Michigan Medical School, Ann Arbor, Michigan, USA; Division of Infectious Diseases, Cedars-Sinai, Los Angeles, California, USA; Department of Obstetrics and Gynecology, Perelman School of Medicine, University of Pennsylvania, Philadelphia, Pennsylvania, USA; Independent Consultant, Rochester, NewYork, USA; Center for Research, Practice and Innovation, Association for Professionals in Infection Control and Epidemiology (APIC), Arlington, Virginia, USA; Page & Page Health, The Ministry, London, UK; Clinical Nurse Consultant, ADVANCING NURSING LLC, Northville, Michigan, USA

**Keywords:** CAUTI, hospital-onset UTI, infection prevention, non-CAUTI, urinary tract infection

## Abstract

**Background:**

Hospital-onset urinary tract infections (HOUTIs), including catheter-associated (CAUTI) and non-catheter-associated UTI (non-CAUTI), remain a significant source of morbidity and healthcare burden. While CAUTI prevention strategies are well established, non-CAUTIs lack standardized definitions, surveillance protocols, and targeted interventions. To address this gap, consensus recommendations identifying prevention strategies for HOUTIs in adult patients were developed.

**Methods:**

A panel of 17 experts in infectious diseases, infection prevention and control, healthcare epidemiology and quality improvement, clinical microbiology, urology, urogynecology, and nursing, participated in a modified Delphi process. An online anonymized survey based on a systematic literature review was completed, before meeting to determine consensus on HOUTI prevention strategies. A further anonymous online survey was shared to finalize recommendations. Thresholds of ≥15/17 panel members in agreement (≥88%) for strong consensus and ≥13/17 (≥76%) for moderate consensus were prospectively set for all statements.

**Results:**

Strong consensus was reached on 37 statements spanning surveillance, intervention selection, strategies and maintenance, related care interventions, specimens and cultures, provider training, and outcome assessment. Key topics—particularly those focused on non-CAUTI HOUTI prevention—were identified as important priorities requiring further exploration and research.

**Conclusions:**

These recommendations offer the foundation for a structured, scalable framework to reduce the burden of all HOUTIs, while also defining future research priorities. By harmonizing established CAUTI best practices with existing literature, and bridging current evidence gaps for non-CAUTI prevention and management with expert consensus, these recommendations provide a road map for improved prevention strategies for all HOUTIs.

## Teaser

A multidisciplinary Delphi panel developed consensus recommendations on preventing all hospital-onset urinary tract infections (HOUTIs)—both catheter-associated UTI (CAUTI) and non-CAUTI—leveraging established CAUTI guidance to close prevention gaps in non-CAUTI and create a scalable framework for comprehensive HOUTI prevention.

Healthcare-associated infections (HAIs) are one of the most common complications of hospital care and represent a significant cause of morbidity and mortality in hospitalized patients [1]. Urinary tract infections (UTIs), which are among the most frequently encountered HAIs [2], likely cost US hospitals in excess of $3.7 billion each year [3].

Indwelling urinary catheters remain one of the most common medical devices employed in emergency departments and hospitals worldwide, and the high frequency of catheter use in hospitalized patients results in a substantial cumulative burden of catheter-associated urinary tract infections (CAUTIs) [4, 5]. Catheter-associated urinary tract infections contribute to prolonged hospital stays, increased antimicrobial resistance, and greater use of healthcare resources. Catheter-associated urinary tract infections have been the target of national and international initiatives to streamline healthcare delivery and improve patient safety, often guided by standardized definitions, surveillance protocols, and evidence-based interventions [4, 6, 7]. However, when assessing clinically meaningful hospital-onset UTIs (HOUTIs), concentrating on CAUTI alone underestimates the true burden and reduces opportunities to prevent all HOUTIs [8].

The latest data from the Centers for Disease Control and Prevention (CDC) indicates that CAUTIs decreased by 11% from 2022 to 2023 [9]. On the other hand, non-CAUTI HOUTIs have been found to be substantially more prevalent than CAUTIs, causing 3-times the number of secondary hospital-onset bacteriemia and fungemia compared with CAUTIs [8]. The management of non-CAUTIs is hindered by a lack of consensus on diagnostic criteria, risk stratification and prevention strategies, impeding accurate surveillance, complicating benchmarking, and perpetuating reactive rather than proactive management. Recent guidance strongly recommends that organizations establish systems to define, analyze, and report non-CAUTI HOUTIs including those associated with alternative devices to indwelling urinary catheters [4, 10]. In spite of those calls for surveillance, the focus of prevention guidance is on CAUTI and indwelling urinary catheters, with few hospitals monitoring non-CAUTI data, and placing minimal focus on the HOUTIs that do not meet the CAUTI definition. Identifying and implementing HAI prevention strategies for both CAUTI and non-CAUTI is therefore a critical gap.

To confront this challenge, we utilized a Delphi approach to identify prevention strategies for all HOUTIs. This work addresses the critical lack of standardized guidance for non-CAUTI management and proposes a structured roadmap for reducing the dual burden of CAUTI and non-CAUTI in adult hospitalized patients. Additionally, it harmonizes existing CAUTI prevention recommendations with existing literature, while also bridging the current evidence gap for non-CAUTI prevention and management with expert consensus into a single set of recommendations.

## METHODS

### Literature Review

Statements and questions were informed by a systematic review of the literature. This review covered relevant observational studies, meta-analyses and randomized controlled trials. This search focused on evidence released since 2013, highlighting the paucity of evidence focused on non-CAUTI HOUTIs. Findings from the literature review were shared with the expert panel members ahead of the Delphi process. This document provided detailed summaries of key outcomes, results, conclusions and study limitations. A comprehensive report detailing the literature review is provided in Supplementary material A.

### Participants

An interdisciplinary panel of 17 experts specializing in infection prevention and control, infectious diseases, healthcare epidemiology and quality improvement, clinical microbiology, urology, urogynecology, and nursing was convened. Participants were selected based on a documented history of publications and clinical practice in their fields, as well as an established professional profile as leading practitioners in a range of clinical and teaching hospitals across the United States. The goal was to ensure balanced representation across diverse settings and specialties.

### Delphi Rounds

For this study, the Delphi consensus method involved a combination of an online survey (Round 1), a hybrid meeting (Round 2), a final online survey containing the refined questions from the previous steps (Round 3), and post-meeting reviews of the consensus results and full manuscript. The questions completed at Round 1 can be found in Supplementary material B. Round 1 was conducted via the SmartSurvey® online platform, and Round 2 was conducted as a hybrid meeting in Chicago, Illinois, United States. E. J. S. acted as Chair, with an independent facilitator (E. S.) moderating the meeting. The Round 3 survey was completed on SmartSurvey®, and panelists were presented with the modified statements from the hybrid meeting. The results following the final survey are shown in Tables 1 and 3. After the results from the Round 3 survey were received, the recommendations and final manuscript were developed, with feedback collected independently from each participant by the facilitator. Responses were collated and updated manuscripts shared at each iteration for individual panel member input and approval. The predefined cutoff for strong consensus was ≥ 15 out of 17 (≥ 88%) panel members in agreement, and for moderate consensus was ≥ 13 out of 18 (≥ 76%) panel members in agreement. Fewer than 12 out of 17 panel members in agreement (71%) was deemed no consensus.

**Table 1.** Summary of Expert Panel Recommendations With Strong Consensus

Number	Statement	Level Of Consensus
	**Surveillance**	
1^a^	It is important to define, monitor and track infection rates using a surveillance definition in units caring for patients with, or at risk of hospital-onset UTIs	100%
2	Non-CAUTI hospital-onset UTI rates should be defined and reported at least monthly	100%
3^a^	Consistent diagnostic criteria and definitions (e.g., positive culture with clinical signs and symptoms) should be used to identify hospital-onset UTIs (both CAUTI and non-CAUTI)	94%
4^a^	The use of electronic systems such as electronic health records (EHRs) is recommended over manual methods for infection surveillance and monitoring	94%
5^a^	EHRs should include documentation of bladder management systems and decision support tools to prompt transitions to less invasive options	94%
6^a^	Identifying the modifiable contributing factors and pathogen responsible for the infection is important to implementing appropriate prevention strategies for all hospital-onset UTIs	94%
	**Intervention selection**	
7^a^	Bladder output measurement should utilize the least invasive devices possible and should become less invasive over time for patients that initially had clinical indications for indwelling catheters	100%
8^a^	Bladder scanners should be routinely used to evaluate urinary retention before considering catheterization for suspected urinary retention	100%
9^b^	Bladder management device selection should be tailored to patient-specific factors (e.g., clinical characteristics, urine output needs, incontinence)	100%
10^a^	Understanding different device types and characteristics and limitations is important in addressing and meeting patient needs	100%
11^a^	To reduce insertion trauma during catheterization attempts, the use of Coudé-tip catheters should be considered in male patients aged over 55 with an enlarged prostate, history of benign prostatic hyperplasia, and/or history of difficult catheterization	94%
12^b^	Bladder scanners, clinical decision support tools, and nurse-driven protocols should be prioritized as strategies to avoid unnecessary catheterization and minimize infection risk	
	Bladder scanners	100%
	Clinical decision support tools	100%
	Nurse-driven protocols per hospital policy	88%
	**Intervention strategies**	
13^b^	Best practices for hand hygiene should be followed by both patients and healthcare workers in order to limit and manage the risk of all hospital-acquired infections	100%
14^a^	Healthcare professional (HCP) adherence to aseptic technique during the insertion of any urinary devices is critical to reduce all hospital-onset UTIs	94%
15^b^	A 2-person/“four-eyes” approach during insertion of a urinary catheter should be considered to reduce infection risk (e.g., in cases of difficult visualization) if possible	94%
16^b^	Sealed, pre-connected closed systems are recommended for appropriate infection control	100%
17	A closed urinary system with a urometer (urimeter, urine meter) should be considered if hourly urine output is anticipated	100%
18^b^	Catheter securement to prevent catheter movement should be employed in all patients with indwelling urinary catheters	100%
19^a^	Insertion location (i.e., ED/OR/ICU/ward) for all patients with an indwelling catheter should be documented to identify areas of opportunity for improvement	100%
	**Intervention maintenance**	
20^a^	Rounding/huddles carried out by the care team are an effective way of ensuring periodic checks are completed regarding the continued need for the current bladder management intervention	100%
21	Patients' bladder management strategies should be reassessed at least daily to prompt the transition of patients to less invasive approaches	94%
22^a^	Perineal hygiene care for patients with or without indwelling urinary catheters should be done with a daily bath and after any episode of diarrhea or fecal incontinence	94%
23	Hygiene for patients with external urinary catheters should be considered at every HCP shift and after any episode of fecal incontinence, diarrhea or indication of device failure	94%
24^b^	Indwelling urinary catheters, and their associated collection systems should be replaced: If the closed system broken or compromised If the catheter is confirmed to be blocked or fails to drain If a urine culture or urinalysis with reflex culture is ordered and a catheter has been in place for >7 days	94% 94% 88%
	**Related care interventions**	
25^a^	Hand hygiene protocols should be strictly followed before and after any interaction with a patient's urinary system or device	100%
	**Specimens and cultures**	
26^b^	Culture stewardship is a key component of high-quality infection control and patient care	100%
27^a^	The use of clean catch, aseptic or sterile techniques in obtaining uncontaminated urine specimens is a key component of diagnostic stewardship	100%
28^a^	Clinician documentation of clinical symptoms should be required before ordering a urine sample	94%
	**Provider training**	
29	Roles and responsibilities for bladder management should be clearly defined across interdisciplinary teams	100%
30^a^	Clinician training should include catheter and alternative urinary device selection, placement, assessment/management, and removal	94%
31^a^	Role-specific training should extend to individuals who may manipulate bladder management systems, including, but not limited to, aids and transport personnel	100%
32^a^	Clinician training should emphasize transitioning to less invasive or alternative bladder management systems	94%
33	Multi-specialty training on hospital-onset UTI prevention, mitigation and diagnostic stewardship should be conducted	94%
34	Role-specific training for clinicians with competency assessment should be completed at the time of hire and annually	88%
	**Outcome assessment**	
35^a^	Infection rates for both CAUTI and non-CAUTI hospital-onset UTIs should be considered a key performance indicator for units/departments managing patients at risk of hospital-onset UTIs	94%
36^a^	Utilization metrics to include alternative bladder management device days should be collected to better understand device utilization	100%
37	The implications of hospital-onset UTI management should be measured with process and outcome metrics such as length of stay, antimicrobial use, device-related trauma, and regression to more invasive devices	100%

^a^Recommendation that expands upon existing practice recommendations [4] by extending guidance to non-CAUTI hospital-onset UTIs, or by extending guidance to interventions beyond indwelling urinary catheters.

^b^Recommendation of the panel that is addressed in existing practice recommendations [4] but is included for completeness of addressing all hospital-onset UTIs.

**Figure 1.**
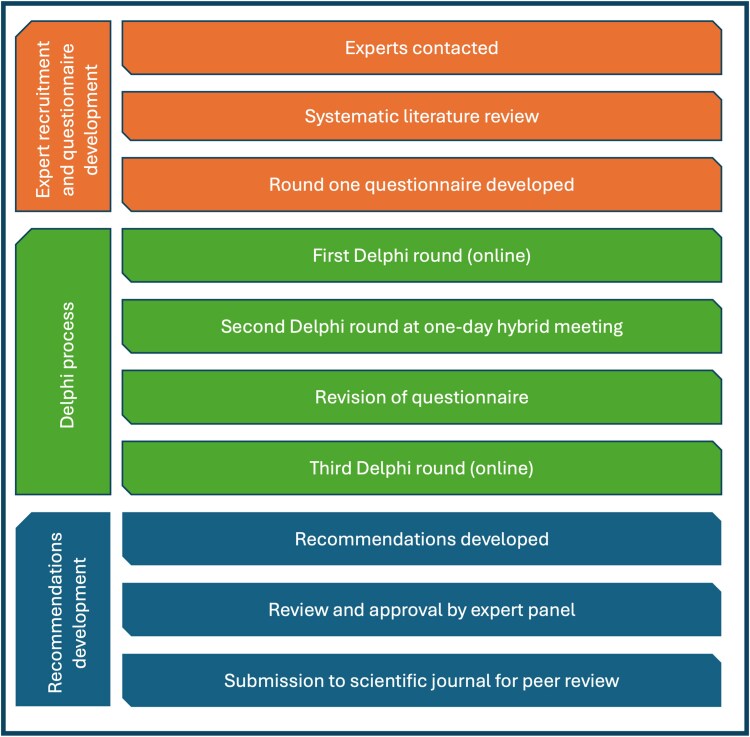
Summary of the Delphi process.

Conflicting opinions were addressed through the structured framework of the Delphi process. The iterative and collaborative approach of the Delphi method fostered a gradual move toward consensus, or, in areas where no consensus was reached, a greater understanding of the key areas for future research. The process for the Delphi panel and subsequent manuscript development is provided in Figure 1.

### Definitions

Discussions during the Delphi process applied specifically to hospitalized adult patients, and discussions centered on all HOUTIs, including both CAUTIs and non-CAUTIs. For the purpose of this work, the following pragmatic definitions from the CDC are proposed [11]:

*CAUTI*: The patient must meet all 3 of the following:

1. Patient had an indwelling urinary catheter that had been in place for more than 2 consecutive days in an inpatient location on the date of event AND was either:
  a. Present for any portion of the calendar day on the date of event

OR

b. Removed the day before the date of event

2. Patient has at least one of the following signs or symptoms:
  - fever (>38.0°C)
  - suprapubic tenderness with no other recognized cause
  - costovertebral angle pain or tenderness with no other recognized cause
  - urinary urgency ^
  - urinary frequency ^
  - dysuria ^

^These symptoms cannot be used when an indwelling urinary catheter (IUC) is in place.

3. Patient has a urine culture with no more than 2 species of organisms identified, at least one of which is a bacterium of ≥10^5^ CFU/ml

*Non-CAUTI*: *The patient must meet all 3 of the following*:

1. One of the following is true:
  a. Patient has/had an indwelling urinary catheter, but it has/had not been in place for more than 2 consecutive days in an inpatient location on the date of event

OR

b. Patient did not have an indwelling urinary catheter in place on the date of event nor the day before the date of event

2. Patient has at least one of the following signs or symptoms:
  - fever (>38°C)
  - suprapubic tenderness with no other recognized cause
  - costovertebral angle pain or tenderness with no other recognized cause
  - urinary frequency ^
  - urinary urgency ^
  - dysuria ^

^These symptoms cannot be used when an indwelling urinary catheter (IUC) is in place.

3. Patient has a urine culture with no more than 2 species of organisms identified, at least one of which is a bacterium of ≥10^5^ CFU/ml.
4. Pyuria:
  - A white blood cell count ≥10/mm^3^.

## RESULTS

The results of the final strong consensus agreements are presented in Table 1, with further detail and rationale provided in Table 2. Eight of the 37 recommendations were unique—recommendations not currently contemplated in the majority of existing practice recommendations [4]. 21 recommendations are extensions to existing practice recommendations [4], either expanding guidance to non-CAUTI HOUTIs or expanding guidance to bladder management interventions beyond indwelling urinary catheters (designated with a “a”). Eight recommendations were very similar existing practice recommendations [4]. They remain in Table 1 for completeness but are noted appropriately (with a “^b^”), to allow for a single point of reference for recommendations addressing all HOUTIs. We elaborate upon unique statements and expanded statements in which strong consensus and moderate consensus were reached in the Areas of Strong Consensus section. To aid adoption of these prevention strategies in real-time practice, a checklist is provided in Supplementary material C.

**Table 2.** Expanded Expert Panel Recommendations With Strong Consensus

**Surveillance**	
1^a^	The panel recommended defining, monitoring and tracking infection rates, noting the risk of over-diagnosing UTIs and excessive antibiotic use which may occur if rates are anchored against inconsistent definitions. The panel recommended setting clinical definitions for CAUTIs and non-CAUTIs, and also surveillance definitions for the purpose of monitoring rates of all HOUTIs. Importantly, and as highlighted by existing evidence [12], the panel emphasized the general lack of evidence focusing on prevention of non-catheter-related infections. In the current absence of evidence-based interventions for these infections, measurement and tracking of infection rates could help to improve clinical understanding of the extent to which these infections are in fact preventable, and which strategies may have greatest impact.
2	Focusing on non-CAUTIs, discussions centered on the importance of defining and internally reporting infection rates, and the frequency of reporting. Forming one of the panel's unique recommendations, monthly reporting of non-CAUTI rates was agreed upon. Granular reporting can be time- and resource-intensive, but this important step aligns with the National Healthcare Safety Network (NHSN) HAI reporting requirements for other infection types [13]. Where resources allow, non-CAUTI surveillance should be integrated into existing HAI prevention programs and conducted at the same frequency as CAUTI reporting.
3^a^	In the absence of effective, evidence-based preventive strategies for non-CAUTIs, the panel stressed the need for consistent diagnostic criteria and definitions. Importantly, such reporting should remain for internal quality improvement purposes only, and not be communicated externally (e.g., with NHSN), as current definitions and diagnostic criteria for non-CAUTIs still require rigorous validation, risk adjustment, and standardization to ensure fair and meaningful comparisons across institutions.
4^a^	The panel focused on the future use of electronic systems such as EHRs versus manual methods for surveillance and monitoring, highlighting the added consistency, reliability, and the reduced burden on clinical resources with the use of electronic methods. Acknowledging the need to match surveillance definitions as closely as possible to clinical definitions, the panel widely agreed that electronic systems were preferred over manual methods, providing the data can be captured and documented in the EHR, and following validation of the EHR measure. The use of electronic systems for documenting cases and identifying populations at risk of UTIs can allow the surveillance and assessment of a greater number of patients, and thus allow researchers to evaluate the impact of UTI prevention interventions, mitigating the need for manual review [14]. The single disagreement reflected concerns about data quality rather than the concept itself. While supportive of EHRs, wholehearted agreement was dependent on the accurate and consistent documentation of all key elements required to inform prevention strategies. Inconsistent or incomplete documentation of key data elements may limit their usefulness for prevention efforts.
5^a^	The panel agreed that having an EHR that allows for the documentation of placement and rationale for a given bladder management system is important, as it facilitates the consideration of, and transition to, less invasive options. Documentation of qualifying UTI symptoms, device placement and removal dates, as well as prompts for timely removal, is also key to effective UTI prevention [15]. Panelists were careful to caveat that prevention efforts are more effective with consistent and reliable documentation of these elements within the EHR.
6^a^	The panel agreed that improving clinical understanding of the modifiable contributing factors and pathogens responsible for infection is key to implementing appropriate prevention strategies for all HOUTIs. Understanding these elements enables targeted antibiotic therapy in appropriate cases, thereby reducing the use of broad-spectrum antibiotics and attendant antimicrobial resistance risk and risk of *Clostridioides difficile* infection. Additionally, recognizing clinical, behavioral and socioecological risk factors can aid the implementation of preventive strategies to reduce recurrence [16].
**Intervention selection**	
7^a^	The panel unanimously aligned with existing literature [4], and agreed that clinical teams should utilize minimally invasive devices to assess patients' bladder output. Alternative devices and less intrusive procedures provide a lower risk of urethral trauma and infection, and can reduce or eliminate noninfectious complications such as discomfort and impaired mobility, and can help to lower healthcare costs [17, 18].
8^a^	In line with existing literature [4], the panel affirmed that bladder scanners should be used to assess and confirm urinary retention to determine the need for both intermittent or indwelling catheterization and to potentially reduce unnecessary catheterization when incomplete bladder emptying is not the cause of the patient's symptoms. The panel suggested bladder scanner volumes sufficient to prompt intermittent catheterization could be ≥300 ml in symptomatic patients, and ≥500 ml in asymptomatic patients [19].
9^†^	In strong support of existing literature [4], the group agreed that the invasiveness of chosen bladder management device should be balanced with the patients' clinical condition and treatment goals.
10^a^	Knowledge of device types and characteristics among clinical teams is important in encouraging customized and patient-centered care, and existing clinical criteria such as the Ann Arbor Criteria for Appropriate Urinary Catheter Use [20] and the Interventions Post-Catheter Removal (iPCaRe) guidance [21] can help to improve care, inform quality improvement efforts, and inform both small- and large-scale interventions for mitigating unnecessary device use.
11^a^	Adding to existing literature surrounding Coudé-tip catheters in male patients, the panel recommended their use in at-risk males, such as men > age 55, those with a diagnosis of benign prostatic hyperplasia, those with an enlarged prostate, and/or a history of difficult catheterization. The panel acknowledged that training levels and availability of these devices can vary, and emphasized that the clinician inserting the catheter should be skilled, and education on its proper insertion and selection should be ongoing.
12^†^	Aligning with existing guidance [4], there was complete consensus on the use of bladder scanners and clinical decision support tools as strategies to avoid unnecessary catheterization and minimization of infection risk. Clinical decision support tools offer an automated, practical and scalable opportunity to target hospital-wide catheter use. Physician-approved, nurse-driven protocols, per hospital policy, were agreed upon as another strategy to enhance patient outcomes and reduce infection rates [17, 22].
**Intervention strategies**	
13^†^	The panel agreed that both patients and healthcare professionals (HCPs) following best practices for hand hygiene can help to mitigate the risk of HAIs. The panel's recommendation strongly supports existing guidance [4].
14^a^	Adherence to aseptic technique during the insertion or application of an intermittent or indwelling urinary catheter by an HCP is important in reducing all HOUTIs. Although the impact of aseptic insertion for indwelling urinary catheters in real-world clinical settings remains unclear [23], it is well established that bacteria can ascend after catheter insertion. As these bacteria may originate from the patient's own flora or the HCPs hands, potentially leading to significant bacteriuria [24], this recommendation adds to existing literature by spanning all HOUTIs.
15^†^	To help maintain optimum aseptic practice and reduce infection risk, the panel discussed the use of a “four-eyes” approach during the insertion of a urinary catheter. Working in pairs was agreed upon as an effective method for minimizing HOUTI risk in line with existing literature [4], especially in certain clinical situations or patient types such as those with difficult visualization of the urethral meatus. The panel acknowledged, however, the challenges in busy or understaffed institutions.
16^†^	Consistent with current guidance [4], the panel recommended the use of a sealed, pre-connected closed catheter system to adhere to infection control best practice. While the quality of evidence is deemed to be low, the maintenance of a sterile, continuously closed catheter drainage system is recommended to reduce catheter-associated bacteriuria and CAUTI in patients with short- and long-term indwelling urethral catheters [4, 12].
17	As another unique recommendation, complete consensus was reached on the pre-emptive selection and use of a closed urinary system with a urometer (urimeter, urine meter), if available, for the monitoring of anticipated hourly urine output. This analytic, precautionary approach mitigates the need to later break the closed system.
18^†^	In accordance with existing guidelines, the panel also agreed that catheter securement to prevent catheter movement should be employed in all patients with indwelling urinary catheters to avoid undue movement or dislodging of the catheter, and to mitigate infection risk, urethral trauma, damage to the bladder neck, pain, and further complications [25].
19^a^	For quality improvement and attribution purposes, the hospital location of device insertion should be documented. Operating Room, Emergency Department, Intensive Care Unit, or ward insertions can vary, both in necessitating reason and in staff-specific practices. The panel agreed that knowing the hospital location in which the device was inserted can better validate placement rationale, more carefully guide appropriate infection prevention and subsequent care strategies, and highlight areas for additional training. Documentation in the EHR is essential to these efforts.
**Intervention maintenance**	
20^a^	There was wide agreement that care team rounding or “huddles” are an effective way of ensuring regular assessment of the ongoing need for the patient's current bladder management intervention. Rounding checklists can provide verification, enhance process standardization and optimize performance improvement [26]. The panel's recommendations align with studies demonstrating that checklists enhance intensive care unit (ICU) best practice compliance by improving interdisciplinary communication [27, 28].
21	Assessment should occur at least daily to understand the reasons for a patients' bladder management strategy, and review the need for continuation [29]. In accordance with current literature, catheter appropriateness should be incorporated into the daily rounding checklist [26], and ongoing assessment will support transitioning patients to progressively less invasive alternatives in line with clinical need and patient characteristics.
22^a^	In patients with or without indwelling urinary catheters, the panel agreed that perineal hygiene care should be done with a daily bath, and following any episode of diarrhea or fecal incontinence to mitigate the risk of all HOUTIs. Research has demonstrated the effectiveness of daily baths with chlorhexidine gluconate (CHG) as a potential strategy to prevent UTIs in the ICU [30]. The panel agreed that daily bathing is an effective strategy for reducing HAIs overall, helping to prevent UTIs when incorporated into a nurse's head-to-toe assessment and incontinence care.
23	There was agreement that hygiene care, including assessment, monitoring and changing, should be considered at least during every HCP shift and after any episode of fecal incontinence or indication of external urinary device failure. The panel noted the differences between male and female external devices, recommending that individual manufacturer specifications should be consulted on indication of device failure and for instructions for changing frequency and maintenance.
24^†^	The frequency of changing indwelling urinary catheters was reviewed with a focus on nuanced scenarios. Strong consensus was reached on changing the indwelling urinary catheter if the closed system is broken or compromised, and if the catheter is confirmed to be blocked or fails to drain. There was also strong consensus that if a urine culture or urinalysis with reflex culture is required and the catheter has been in place for 7 days or more, it should be changed before obtaining the sample. These recommendations are consistent with current practice recommendations [4].
**Related care interventions**	
25^a^	The panel reached complete consensus on the strict adherence to hand hygiene protocols during any interaction with a patient's bladder management device. This aligns with current literature, guidelines, and clinical practice [4].
**Specimens and cultures**	
26^†^	In line with current evidence [4], the panel agreed that urine culture stewardship is a key aspect of high-quality antimicrobial stewardship and patient care, ensuring optimized use of urine cultures when indicated to guide antibiotic therapy [31]. Urine culture stewardship is applicable across diverse clinical settings and is relevant for patients both with and without indwelling urinary catheters, helping not only to reduce CAUTI prevalence, but also the number of asymptomatic bacteriuria (ASB) cases treated inappropriately [31]. Education for HCPs around urine culture stewardship should be encouraged, with an emphasis on reviewing appropriate indications before ordering [4].
27^a^	Clean catch, sterile or aseptic techniques for obtaining urine samples was deemed a key component of proper diagnostic stewardship by the panel, extending existing practice recommendations beyond simply disinfecting the tubing sample port on patients managed with indwelling urinary catheters [4]. The collection and handling of urine cultures affect both the accuracy of surveillance for infection and antibiotic stewardship [32]. In line with current literature, the panel agreed that a mid-stream clean catch method with cleansing is an effective way to reduce risk of contamination [33, 34].
28^a^	Before ordering a urine sample, the panel emphasized the importance of clinicians documenting clinical symptoms. However, uncertainty remained about which signs and symptoms were appropriate, and the importance of optimizing test ordering and downstream antibiotic treatment were emphasized. Proper ordering of urine cultures is crucial in cases where screening and treatment of ASB are recommended. These include pregnant women and individuals undergoing urologic procedures associated with mucosal trauma [35], as screening and treatment in these populations help lower the risk of post-procedure infections.
**Provider training**	
29	Complete consensus was reached on roles and responsibilities being clearly defined across interdisciplinary teams overseeing patients' bladder management with emphasis on bladder management covering interventions beyond simply indwelling urinary catheters.
30^a^	The panel agreed that clinician training is crucial and should cover competencies on indwelling urinary catheter and alternative urinary device selection, placement, assessment, management, and removal to ensure best practices [36].
31^a^	The panel highlighted the need for training all individuals involved in bladder management system manipulation, including, but not limited to, assistive and transport personnel. Additionally, rapid transport and processing of urine samples, adherence to aseptic technique during indwelling catheter insertion, and ensuring transport personnel maintain proper catheter positioning during patient transfers or manipulations were cited as areas for attention.
32^a^	Additionally, a migration toward less or noninvasive bladder management systems should be encouraged among all clinical staff, through education and real-time learning reinforcement.
33	Training and competency assessment should occur across multiple disciplines, including, but not limited to, physicians, nurses, urology and infection control professionals, focusing on diagnostic stewardship for HOUTI prevention and mitigation. The panel emphasized the recommendation for ongoing training and professional development to maintain staff skills in infection control and prevention in line with current evidence [37].
34	The panel recommended that role-specific training on prevention, diagnosis and management of all HOUTIs be done at the time of hire and then annually, or as indicated. Training physicians on the known complications of each bladder management device is important, along with guidance on appropriate intervention selection, and timing of assessment and discontinuation. As nursing staff are typically responsible for device placement, it is essential to provide role-specific training to ensure their skills are maintained to optimize patient safety.
**Outcome assessment**	
35^a^	There was agreement that departments managing patients at risk for hospital-onset UTIs should consider rates for both CAUTI and non-CAUTI hospital-onset UTI as a key performance indicator for internal reporting and benchmarking purposes as well as process improvement. The panel was careful to note that these performance measures may not directly meet external reporting obligations. Currently, this is especially important in non-CAUTI cases, given the absence of validated measures and definitions and evidence-based preventive guidelines.
36^a^	Utilization metrics including the device days associated with alternative bladder management devices should be collected to allow clinical teams to better understand utilization of all bladder management devices. If non-CAUTI surveillance is performed, alternative device utilization should be determined. For CAUTI specifically, the device utilization ratio (DUR) can be used as a performance measure to address potential indwelling catheter-associated harm. The DUR is patient-centered, objective, and is currently captured as an aspect of NHSN reporting [38].
37	Complete consensus was reached on the importance of evaluating the management of HOUTIs through process and outcome metrics such as length of stay, antimicrobial usage, device-related trauma, and the transition to less invasive devices. Measurement of these metrics was deemed crucial for enhancing patient care, optimizing resources, and guiding quality improvement and stewardship efforts. The panel noted that systematically tracking these metrics can inform targeted interventions through the identification of preventable harms, helping to reduce infection rates, minimization of device-related complications, and promotion of appropriate antimicrobial use.

^a^Recommendation that expands upon existing practice recommendations [4] by extending guidance to non-CAUTI hospital-onset UTIs, or by extending guidance to interventions beyond indwelling urinary catheters.^†^Recommendation of the panel that is addressed in existing practice recommendations [4] but is included for completeness of addressing all hospital-onset UTIs.

Topics on which no consensus was reached also provide an important outcome from this work, with discussions helping to identify valuable areas for further research. These topics are also expanded upon in the Areas Identified for Future Research section, and Supplementary material D. Several topics achieved moderate consensus, but these topics largely echo themes already elaborated upon in the Areas of Strong Consensus section. To acknowledge their importance without overburdening the main narrative, we have compiled these moderately agreed-upon items in Supplementary material E.

### Areas Identified for Future Research

Through the iterative rounds of the Delphi process, several key areas were identified as important areas for future research, and are presented in Table 3. These topics remained unresolved after multiple rounds of voting and extensive discussion. In all cases, topics were discussed in detail. However, in some cases, statements were discussed by the panel but not subjected to voting as they were deemed to fall within practices that require further research. These topics are denoted by “topic discussed, not voted upon” in Table 3. For other statements, voting was conducted but consensus was not reached, and the panel agreed that they too require further research.

**Table 3.** Areas Identified for Future Research

Number	Topic	Level Of Consensus
	**Intervention selection**	
1	The initiation of additional preventive interventions such as bladder care protocols and hydration support should be considered at admission in at-risk patients	No consensus
2	Antimicrobial catheters should be used in all patients	Topic discussed, not voted upon
	**Intervention strategies**	
3	Prior to the insertion of all intraurethral bladder management interventions e.g., indwelling urinary catheters and intermittent catheters, antiseptic solutions should be used for cleaning the urethral meatus	Topic discussed, not voted upon
	**Intervention maintenance**	
4	Non-suction-assisted urine collection systems should be placed below the level of the bladder	Topic discussed, not voted upon
5	A sealed, preconnected closed drainage system should be maintained for patients with indwelling urinary catheters, excluding suprapubic catheters, to reduce infection risk	No consensus
6	Female external suction catheters should be replaced: Every 8 h Every 12 h Every HCP shift Every 24 h When soiled or otherwise indicated Other	Topic discussed, not voted upon
7	Male external suction catheters should be replaced: Every 8 h Every 12 h Every HCP shift Every 24 h When soiled or otherwise indicated Other	Topic discussed, not voted upon
8	Male external condom catheters should be replaced: Every 8 h Every 12 h Every HCP shift Every 24 h When soiled or otherwise indicated Other	Topic discussed, not voted upon
	**Related care interventions**	
9	Bathing/treatment with specific solutions should be performed to reduce the risk of all hospital-onset UTIs	Topic discussed, not voted upon
10	Targeted decolonization (e.g., skin antiseptics) plays a key role in reducing UTI risk	Topic discussed, not voted upon
	**Specimens and cultures**	
11	In symptomatic* patients with and without indwelling urinary catheters, it is appropriate to use urinalysis with reflex to culture as a screening tool *Symptomatic patients include those with fever, flank pain, pelvic discomfort, altered mental status with symptoms of infection e.g., fever/temperature >100.4°F	No consensus
12	The adoption of molecular diagnostic techniques such as polymerase chain reaction-based pathogen identification on urine is important in improving the speed and accuracy of UTI diagnosis in hospitalized patients	No consensus
	**Outcome assessment**	
13	Hospitals should establish surveillance protocols and begin reporting non-catheter associated hospital-onset UTIs through the NHSN to allow performance improvement interventions to be conducted	No consensus

A lack of high-quality evidence was a barrier to arriving at consensus in several areas that may mitigate non-CAUTI HOUTIs. Those areas included hydration, antiseptic cleaning of the urethral meatus, bathing/treatment with antiseptic solution, targeted decolonization of patients, adoption of molecular diagnostic testing, reporting non-catheter associated HOUTIs through the NHSN (Topics 1, 3, 9, 10, 12, and 13). A paucity of recent favorable research was a challenge for the area of antimicrobial catheters (Topic 2). Existing guidance was felt to be sufficient for the areas of system placement below the level of the collection bag (Topic 4). A number of areas involving the timing of external device replacement were deemed best left to manufacturers' instructions (Topics 6, 7, and 8). The complexity of the topic and the need for more granular guidance challenged consensus in the areas of the maintenance of appropriate drainage systems for suprapubic catheters (Topic 5) and urinalysis with reflex to culture as a screening tool (Topic 11). Detailed descriptions of the panel's analysis of these thirteen topics are supplied in Supplementary material D.

## DISCUSSION

This Delphi consensus panel project provides comprehensive recommendations for the prevention of all HOUTIs, including both CAUTI and non-CAUTI. For CAUTI, the panel reaffirmed established strategies such as prompt catheter removal, closed drainage systems, aseptic insertion, and antibiotic stewardship, reflecting a mature body of evidence and alignment with current guidance.

By contrast, the panel's most distinctive contribution lies in advancing guidance for non-CAUTIs, which is an area with limited data, variable prevention bundles, and inconsistent diagnostic criteria. Monthly internal reporting of non-CAUTI rates was uniquely recommended, given the absence of validated benchmarks for external comparison with emphasis on standardized clinical definitions. More broadly, the panel highlighted the importance of diagnostic and culture stewardship, with role-specific training extending to aids and transport personnel, use of electronic health records, and careful documentation of symptoms as central to both appropriate diagnosis and antimicrobial stewardship in these patients.

Finally, panel consensus emphasized ongoing assessment of bladder management system necessity (not limited to indwelling urinary catheters), incorporation of minimally invasive alternatives, and systematic tracking of broader outcome measures including device days (importantly, alternative device days as well as indwelling urinary catheter days), antimicrobial exposure and noninfectious harms beyond infection rates alone. In summary, while CAUTI prevention strategies remain grounded in long-established practices, this panel calls for renewed attention to the under-recognized burden of non-CAUTIs, underscoring the urgent need for validated definitions, standardized surveillance, and a focused research agenda to inform meaningful prevention efforts.

The Delphi technique is a systematic process of gathering collective opinion of knowledgeable panel members [39]. It offers several advantages, including anonymity in the survey rounds, controlled feedback, and iterative discussions [39]. It encourages participants to reflect and potentially revise their views as discussions progress, bringing a depth of consideration that is often missing from other information gathering methods such as advisory boards, interviews or focus groups [40]. Anonymity during survey stages helps minimize social bias and supports candid responses, while the structured, multi-round nature of the process provides a dynamic and adaptable way to capture expert perspectives on specific research topics. This iterative method enables the anonymous input of contributors from a wide range of locations and specialties, reducing the influence of dominant voices. Through voting rounds, it serves as a robust strategy for reaching expert consensus, especially in areas lacking strong evidence-based interventions and where expert opinion is valued [41, 42]. In this study, a modified Delphi format was used, blending the initial structured collection of anonymous feedback with hybrid (in-person and virtual) discussion and follow-up voting rounds to assess consensus on prevention strategies for all HOUTIs. To mitigate the risk of superficial or group consensus, each statement was accompanied by vigorous discussion, allowing panelists to articulate reservations, challenge assumptions, and thoughtfully reconsider their responses across rounds.

While it has clear strengths, the Delphi approach is not without limitations. It can be affected by participant attrition and unrealized survey gaps. Selection bias and failure to include all relevant stakeholders can potentially constrict the scope of recommendations despite best efforts to recruit a diverse panel of stakeholders. In addition, social dynamics during the meeting may have shaped collective responses, and the balance of input from remote versus in-person attendees may not have been even. Lastly, panelists were reluctant at times to advance expert opinion in areas where evidence was thin—especially those practices associated with non-CAUTI HOUTIs.

## CONCLUSIONS

Hospital-onset UTIs remain a persistent and costly complication of inpatient care. Although decades of focused intervention have significantly reduced CAUTI rates, non-CAUTI HOUTIs continue to impose severe consequences, including secondary bacteriemia and fungemia, increased antimicrobial use, and elevated resource utilization. Despite their prevalence and impact, non-CAUTI HOUTIs remain poorly defined, lacking clear diagnostic criteria, standardized surveillance protocols, and evidence-based prevention bundles, leaving a significant gap in hospital infection control efforts. In the absence of professional society guidelines for non-CAUTI prevention, we aimed to develop and identify interim strategies. By convening an interdisciplinary expert panel, we harmonized established CAUTI prevention best practices with many novel, consensus-driven recommendations tailored to non-CAUTI HOUTIs. This process yielded a structured framework for standardized definitions, risk stratification, and evidence-informed interventions applicable across all HOUTIs.

Our findings highlight the importance of a staged implementation strategy, beginning with robust surveillance and data aggregation, aimed at identifying and mitigating unnecessary catheterization, and transitions to progressively less invasive bladder management alternatives, with the goal of reducing both CAUTI and non-CAUTI HOUTI rates. Ultimately, this comprehensive approach not only identifies key evidence gaps but also establishes a practical and scalable roadmap for closing the critical knowledge gap in HAI prevention. The areas of moderate consensus and future research were predominately related to non-CAUTI HOUTIs, emphasizing the need for evidence generation surrounding practices that look beyond simply CAUTIs. The framework outlined here aims to drive meaningful change and reduce the dual burden of CAUTI and non-CAUTI HOUTIs in adult hospitalized patients. Addressing these challenges is essential to enhancing patient safety, improving outcomes, and advancing infection prevention efforts in the acute care setting.

## Supplementary Material

ofag060_Supplementary_Data

## Supplementary Data

Supplementary materials are available at *Open Forum Infectious Diseases* online. Consisting of data provided by the authors to benefit the reader, the posted materials are not copyedited and are the sole responsibility of the authors, so questions or comments should be addressed to the corresponding author.

## References

[1] Organisation for Economic Co-operation and Development (OECD) . Addressing the Burden of Infections and Antimicrobial Resistance Associated with Health Care. **2022** Available at: https://www.oecd.org/content/dam/oecd/en/topics/policy-sub-issues/antimicrobial-resistance-and-pandemics/addressing-burden-of-infections-and-amr-associated-with-health-care.pdf. Last accessed March 2025.

[2] Patel R, Polage CR, Dien Bard J, et al Envisioning future urinary tract infection diagnostics. Clin Infect Dis 2022; 74:1284–92. 34463708 10.1093/cid/ciab749 PMC8994576

[3] Scott RD 2nd, Culler SD, Baggs J, et al Measuring the direct medical costs of hospital-onset infections using an analogy costing framework. Pharmacoeconomics. 2024; 42:1127–44. 38967909 10.1007/s40273-024-01400-z PMC11405445

[4] Patel PK, Advani SD, Kofman AD, et al Strategies to prevent catheter-associated urinary tract infections in acute-care hospitals: 2022 update. Infect Control Hosp Epidemiol 2023; 44:1209–31. 37620117 10.1017/ice.2023.137 PMC11287483

[5] Saint S, Chenoweth CE. Biofilms and catheter-associated urinary tract infections. Infect Dis Clin North Am 2003; 17:411–32. 12848477 10.1016/s0891-5520(03)00011-4

[6] Meddings J, Reichert H, Greene MT, et al Evaluation of the association between Hospital Survey on Patient Safety Culture (HSOPS) measures and catheter-associated infections: results of two national collaboratives. BMJ Qual Saf 2017; 26:226–35. 10.1136/bmjqs-2015-005012 PMC5122467 27222593

[7] Rosenthal VD, Memish ZA, Nicastri E, et al Preventing catheter-associated urinary tract infections: a position paper of the International Society for Infectious Diseases, 2024 update. Int J Infect Dis 2025; 151:107304. 39551089 10.1016/j.ijid.2024.107304

[8] Kelly T, Ai C, Jung M, Yu K. Catheter-associated urinary tract infections (CAUTIs) and non-CAUTI hospital-onset urinary tract infections: relative burden, cost, outcomes and related hospital-onset bacteremia and fungemia infections. Infect Control Hosp Epidemiol 2024; 45:864–71. 38374686 10.1017/ice.2024.26 PMC11439594

[9] Centers for Disease Control and Prevention (CDC) . Current HAI Progress Report. **2024**. Available at: https://www.cdc.gov/healthcare-associated-infections/php/data/progress-report.html. Last accessed April 2025.

[10] Crapanzano-Sigafoos R, Phillips B, Ormsby J, et al Guide to preventing catheter-associated urinary tract infections (CAUTI). Arlington, VA: The Association for Professionals in Infection Control and Epidemiology (APIC), 2025.

[11] Centers for Disease Control and Prevention (CDC) . Urinary tract infection (catheter-associated urinary tract infection [CAUTI] and non-catheter-associated urinary tract infection [UTI]) events. **2025** Available at: https://www.cdc.gov/nhsn/pdfs/pscmanual/7psccauticurrent.pdf. Last accessed June 2025.

[12] Hooton TM, Bradley SF, Cardenas DD, et al Diagnosis, prevention, and treatment of catheter-associated urinary tract infection in adults: 2009 International Clinical Practice Guidelines from the Infectious Diseases Society of America. Clin Infect Dis 2010; 50:625–63. 20175247 10.1086/650482

[13] Centers for Disease Control and Prevention (CDC) . Identifying Healthcare-associated Infections (HAI) for NHSN surveillance. **2025** Available at: https://www.cdc.gov/nhsn/pdfs/pscmanual/2psc_identifyinghais_nhsncurrent.pdf. Last accessed March 2025.

[14] Colborn KL, Bronsert M, Hammermeister K, et al Identification of urinary tract infections using electronic health record data. Am J Infect Control 2019; 47:371–5. 30522837 10.1016/j.ajic.2018.10.009 PMC6312639

[15] Werneburg GT . Catheter-associated urinary tract infections: current challenges and future prospects. Res Rep Urol 2022; 14:109–33. 35402319 10.2147/RRU.S273663 PMC8992741

[16] American Urological Association . Recurrent Uncomplicated Urinary Tract Infections in Women: AUA/CUA/SUFU Guideline (2022). **2022** Available at: https://www.auanet.org/guidelines-and-quality/guidelines/recurrent-uti. Last accessed March 2025.

[17] Agency for Healthcare Research & Quality . Toolkit for Reducing Catheter-Associated Urinary Tract Infections in Hospital Units: Implementation Guide. **2015** Available at: https://www.ahrq.gov/sites/default/files/publications/files/implementation-guide_0.pdf. Last accessed March 2025.

[18] Michigan Surgical Quality Collaborative (MSQC) . Alternatives to Indwelling Urinary Catheter Use. Available at: https://www.msqc.org/_files/ugd/d5fc0a_e53c007376e9400899e1fcb5bdaef251.pdf. Last accessed March 2025.

[19] Chrouser K, Fowler KE, Mann JD, et al Urinary retention evaluation and catheterization algorithm for adult inpatients. JAMA Netw Open 2024; 7:e2422281. 39012634 10.1001/jamanetworkopen.2024.22281 PMC11252892

[20] Meddings J, Saint S, Fowler KE, et al The Ann Arbor criteria for appropriate urinary catheter use in hospitalized medical patients: results obtained by using the RAND/UCLA appropriateness method. Ann Intern Med 2015; 162:S1–S34. 25938928 10.7326/M14-1304

[21] Gray M, Beeson T, Kent D, et al Interventions Post Catheter Removal (iPCaRe) in the acute care setting: an evidence-and consensus-based algorithm. J Wound Ostomy Continence Nurs 2020; 47:601–18. 33201147 10.1097/WON.0000000000000704

[22] Su L . Effectiveness of nurse-driven protocols in reducing catheter-associated urinary tract infections: a systematic review and meta-analysis. J Nurs Care Qual 2025; 40:39–45. 39418341 10.1097/NCQ.0000000000000811

[23] Manojlovich M, Saint S, Meddings J, et al Indwelling urinary catheter insertion practices in the emergency department: an observational study. Infect Control Hosp Epidemiol 2016; 37:117–9. 26434781 10.1017/ice.2015.238

[24] Felix K, Bellush MJ, Bor B, Greene L, ed. Apic implementation guide: guide to preventing catheter-associated urinary tract infections (CAUTIs). Washington: Association for Professionals in Infection Control and Epidemiology; 2014. Available at: https://www.mghpcs.org/eed/cau/Assets/documents/cau/APIC-Implementation-Guide.pdf. Last accessed February 2025.

[25] Yates A . The importance of fixation and securing devices in supporting indwelling catheters. Br J Community Nurs 2013; 18:588–90. 24335791 10.12968/bjcn.2013.18.12.588

[26] Nassikas NJ, Monteiro JF, Pashnik B, et al Intensive care unit rounding checklists to reduce catheter-associated urinary tract infections. Infect Control Hosp Epidemiol 2020; 41:680–3. 32127059 10.1017/ice.2020.43

[27] Carlos WG, Patel DG, Vannostrand KM, et al Intensive care unit rounding checklist implementation. Effect of accountability measures on physician compliance. Ann Am Thorac Soc 2015; 12:533–8. 25642750 10.1513/AnnalsATS.201410-494OC

[28] Centofanti JE, Duan EH, Hoad NC, et al Use of a daily goals checklist for morning ICU rounds: a mixed-methods study. Crit Care Med 2014; 42:1797–803. 24674928 10.1097/CCM.0000000000000331

[29] Royal College of Nursing . Catheter Care. RCN Guidance for Health Care Professionals. **2021** Available at: https://www.nhsggc.scot/downloads/rcn-catheter-care-guidelines-2021/. Last accessed April 2025.

[30] Huang SS, Septimus E, Hayden MK, et al Effect of body surface decolonisation on bacteriuria and candiduria in intensive care units: an analysis of a cluster-randomised trial. Lancet Infect Dis 2016; 16:70–9. 26631833 10.1016/S1473-3099(15)00238-8

[31] Centers for Disease Control and Prevention (CDC) . Indwelling Urinary Catheter Culture Stewardship: Overview. **2024** Available at: https://www.cdc.gov/uti/hcp/clinical-guidance/index.html#:∼:text=a%20urine%20sample.-, Multifaceted%20approach,have%20bacteriuria%20after%20one%20month. Last accessed May 2025.

[32] Garcia R, Spitzer ED. Promoting appropriate urine culture management to improve health care outcomes and the accuracy of catheter-associated urinary tract infections. Am J Infect Control 2017; 45:1143–53. 28476493 10.1016/j.ajic.2017.03.006

[33] Hansen MA, Valentine-King M, Zoorob R, et al Prevalence and predictors of urine culture contamination in primary care: a cross-sectional study. Int J Nurs Stud 2022; 134:104325. 35914376 10.1016/j.ijnurstu.2022.104325 PMC10513105

[34] LaRocco MT, Franek J, Leibach EK, et al Effectiveness of preanalytic practices on contamination and diagnostic accuracy of urine cultures: a laboratory medicine best practices systematic review and meta-analysis. Clin Microbiol Rev 2016; 29:105–47. 26598386 10.1128/CMR.00030-15 PMC4771218

[35] Nicolle LE, Gupta K, Bradley SF, et al Clinical practice guideline for the management of asymptomatic bacteriuria: 2019 update by the Infectious Diseases Society of America. Clin Infect Dis 2019; 68:e83–e110. 30895288 10.1093/cid/ciy1121

[36] Centers for Disease Control and Prevention (CDC) . Guideline for Prevention of Catheter-Associated Urinary Tract Infections (2009). Summary of recommendations. **2009**. Available at: https://www.cdc.gov/infection-control/hcp/cauti/summary-of-recommendations.html. Last accessed March 2025.

[37] Zhang M, Wu S, Ibrahim MI, et al Significance of ongoing training and professional development in optimizing healthcare-associated infection prevention and control. J Med Signals Sens 2024; 14:14. 39100741 10.4103/jmss.jmss_37_23 PMC11296567

[38] Fakih MG, Gould CV, Trautner BW, et al Beyond infection: device utilization ratio as a performance measure for urinary catheter harm. Infect Control Hosp Epidemiol 2016; 37:327–33. 26894622 10.1017/ice.2015.287 PMC6502466

[39] Nasa P, Jain R, Juneja D. Delphi methodology in healthcare research: how to decide its appropriateness. World J Methodol 2021; 11:116. 34322364 10.5662/wjm.v11.i4.116 PMC8299905

[40] Barrett D, Heale R. What are Delphi studies? Evid Based Nurs 2020; 23:68–9. 32430290 10.1136/ebnurs-2020-103303

[41] Meshkat B, Cowman S, Gethin G, et al Using an e-Delphi technique in achieving consensus across disciplines for developing best practice in day surgery in Ireland. J Hosp Adm 2014; 3:1.

[42] Eubank BH, Mohtadi NG, Lafave MR, et al Using the modified Delphi method to establish clinical consensus for the diagnosis and treatment of patients with rotator cuff pathology. BMC Med Res Methodol 2016; 16:1–5. 27206853 10.1186/s12874-016-0165-8 PMC4875724

