# Supplementary material for: Prevention Strategies for All Hospital-Onset Urinary Tract Infections: Best Practice Consensus Recommendations
Source: Open Forum Infect Dis. 2026 Feb 6;13(2):ofag060. doi: 10.1093/ofid/ofag060 (PMC12919440; doi:10.1093/ofid/ofag060)
Supplement: ofag060_Supplementary_Data [file ofag060_supplementary_data.zip › Supplementary material C_UTI prevention_Checklist.docx]

**Hospital-Onset Urinary Tract Infection (UTI) prevention checklist**

**Consensus recommendations for preventing catheter-associated UTIs (CAUTIs) and non-CAUTI hospital-onset UTIs**

| **Selection** |
| --- |
| Utilize the least invasive devices possible, with those interventions becoming less invasive over time for patients that initially had clinical indications for indwelling catheters |
| Routinely use bladder scanners to evaluate urinary retention before considering catheterization for suspected urinary retention |
| Tailor bladder management device selection to patient-specific factors (e.g., clinical characteristics, urine output needs, incontinence) |
| Understand the different device types and characteristics and limitations – this is important toward addressing and meeting patient needs |
| Consider the use of Coudé-tip catheters in male patients aged over 55 with an enlarged prostate, history of benign prostatic hyperplasia, and/or history of difficult catheterization to reduce insertion trauma during catheterization attempts |
| Prioritize strategies to avoid unnecessary catheterization and minimize infection risk:   - Bladder scanners - Clinical decision support tools - Nurse-driven protocols per hospital policy |
| **Insertion/Application** |
| Follow best practices for hand hygiene for both patients and healthcare providers (HCPs) in order to limit and manage the risk of all hospital-acquired infections |
| Adhere to aseptic technique during the insertion of any urinary devices – this is critical to reduce all hospital-onset UTIs |
| Consider a two-person / ‘four eyes’ approach during insertion of a urinary catheter to reduce infection risk (e.g. in cases of difficult visualisation) if possible |
| Employ sealed, pre-connected closed systems for appropriate infection control |
| Utilize a closed urinary system with a urometer (urimeter, urine meter) if hourly urine output is anticipated |
| Employ catheter securement to prevent catheter movement in all patients with indwelling urinary catheters |
| Document insertion location (i.e., ED / OR / ICU / ward) for all patients with an indwelling catheter to identify areas of opportunity for improvement |
| **Maintenance** |
| Consider rounding/huddles carried out by the care team to ensure periodic checks are completed regarding the continued need for the current bladder management intervention |
| Reassess patients’ bladder management strategies at least daily to prompt the transition of patients to less invasive approaches |
| Perform perineal hygiene care for patients with or without indwelling urinary catheters with a daily bath and after any episode of diarrhea or fecal incontinence |
| Perform hygiene for patients with external urinary catheters at every HCP shift change and after any episode of fecal incontinence, diarrhea or indication of device failure |
| Replace indwelling urinary catheters, and their associated collection systems:   - If the closed system broken or compromised - If the catheter is confirmed to be blocked or fails to drain - If a urine culture or urinalysis with reflex culture is ordered and a catheter has been in place for >7 days |
| **Related care** |
| Strictly follow hand hygiene protocols before and after any interaction with a patient’s urinary system or device |

**Hospital-Onset Urinary Tract Infection (UTI) prevention checklist**

**Consensus recommendations for preventing catheter-associated UTIs (CAUTIs) and non-CAUTI hospital-onset UTIs**

| **Specimens and cultures** |
| --- |
| Diagnostic stewardship is a key component of high-quality infection control and patient safety |
| Use clean catch, aseptic or sterile techniques in obtaining uncontaminated urine specimens |
| Before ordering a urine sample, ensure clinician documentation of clinical symptoms or indication |
| **Provider training** |
| Clearly define roles and responsibilities for bladder management across interdisciplinary teams |
| Ensure that clinician training includes catheter and alternative urinary device selection, placement, assessment/management, and removal |
| Extend role-specific training to individuals who may manipulate bladder management systems, including, but not limited to, aids and transport personnel |
| Emphasize transitioning to less invasive or alternative bladder management systems with clinician training |
| Conduct multi-specialty training on hospital-onset UTI prevention, mitigation and diagnostic stewardship |
| Complete role-specific training for clinicians with competency assessment at the time of hire and annually |
| **Surveillance** |
| Define, monitor and track infection rates using a surveillance definition in units caring for patients with, or at risk of hospital-onset UTIs |
| Define and report non-CAUTI hospital-onset UTI rates at least monthly |
| Use consistent diagnostic criteria and definitions (e.g., positive culture with clinical signs and symptoms) to identify hospital-onset UTIs (both CAUTI and non-CAUTI) |
| Use electronic systems such as electronic health records (EHRs) over manual methods for infection surveillance and monitoring |
| Include documentation of bladder management systems and decision support tools to within the EHR to prompt transitions to less invasive options |
| Identify the modifiable contributing factors and pathogen responsible for the infection – this is important to implement appropriate prevention strategies for all hospital-onset UTIs |
| **Outcome assessment** |
| Consider infection rates for both CAUTI and non-CAUTI hospital-onset UTIs as key performance indicators for units / departments managing patients at risk of hospital-onset UTIs |
| Collect utilization metrics to include alternative bladder management device days to better understand device utilization |
| Measure the implications of hospital-onset UTI management with process and outcome metrics such as length of stay, antimicrobial use, device-related trauma, and regression to more invasive devices |
