# Supplementary material for: Prevention Strategies for All Hospital-Onset Urinary Tract Infections: Best Practice Consensus Recommendations
Source: Open Forum Infect Dis. 2026 Feb 6;13(2):ofag060. doi: 10.1093/ofid/ofag060 (PMC12919440; doi:10.1093/ofid/ofag060)
Supplement: ofag060_Supplementary_Data [file ofag060_supplementary_data.zip › Supplementary material D_Topics for future research_v2 with references.docx]

**Discussion on areas identified for future research**

**Intervention selection**

**Topics 1–2**

The panel acknowledged that hydration support as a preventive measure is an area that has been more extensively studied in the long-term geriatric literature, ^1^ with a relative lack of evidence to support recommendations in the acute care setting. Nursing home evidence highlights the negative impact that inadequate fluid intake can have on urine osmolarity and frequency of voiding, both of which are believed to be factors in bacterial growth. ^2^ It is acknowledged that adequate hydration is important for patients at risk of UTIs. However, the panel acknowledged the heterogeneity of different patient populations, particularly for patients with congestive heart failure, where hydration strategies must be tailored to individual clinical needs. Given these complexities, the panel determined that confirming hydration support as a definitive HOUTI prevention strategy was beyond its scope.

The panel was unable to reach consensus on the routine use of antimicrobial catheters for all patients, citing a lack of sufficient recent evidence and guidance to support their efficacy and safety in preventing CAUTI. According to current evidence, silver-coated catheters are the most popular and widely tested catheters among bactericidal-coated catheters, ^3^ and although older research has shown these devices to be effective, ^4,5^ more recent studies have presented mixed findings. ^6,7^ The panel noted that the latest Infectious Diseases Society of America (IDSA) Compendium of Strategies Update do not recommend the routine use of antimicrobial catheters, ^8^ and while there is some evidence to support the reduced risk of CAUTI in patients requiring longer-term catheters, ^3^ any benefit among patients requiring long-term catheterization should be balanced against the increased direct costs to health systems when compared to non-coated catheters. ^3^ Current research remains inconclusive, highlighting the need for further studies to establish definitive recommendations.

**Intervention strategies**

**Topic 3**

The panel discussed the use of antiseptic solutions for cleaning the urethral meatus and aligned to current guidance, ^8^ which states that it is a currently unresolved issue. While recent CAUTI prevention guidance from the Association for Professionals in Infection Control and Epidemiology (APIC) highlights the potential benefits of using a chlorhexidine gluconate (CHG)-impregnated cloth to cleanse a portion of the indwelling urinary catheter, ^9^ little evidence exists to support the use of an antiseptic solution to cleanse the urethral meatus specifically, so it was deemed beyond the scope of the panel to recommend its use. The panel did however discuss the link between prior insertion of a urinary catheter and the development of non-CAUTI HOUTI and emphasized the importance of aseptic catheter care in reducing that risk.

**Intervention maintenance**

**Topics 4–8**

Per current evidence and guidance, ^8,10^ the panel agreed that an unobstructed urine flow should be maintained with indwelling urinary catheters, with guidance stating that collecting bags should be kept below the level of the bladder and kept free from kinking to avoid infection risk. For external collection devices, it was recommended that practitioners refer to individual manufacturer guidance.

While the panel previously recommended the use of a sealed, pre-connected closed system for appropriate infection control aligning with current guidance, ^8,11^ they were unable to reach consensus regarding those patients with suprapubic catheters. The panel agreed that having a prior urinary catheter is a likely risk factor for developing HOB secondary to a non-CAUTI HOUTI, but there was disagreement about whether this risk extends to longer-term suprapubic catheters. Given that the published literature already includes detailed guidance on the indications and use of suprapubic catheters, such as in cases of acute retention with prostatitis or post-genitourinary surgery, the panel felt it was unnecessary to issue further recommendations.

Regarding the replacement of female and male external suction catheters, as well as male external (condom) catheters, the panel emphasized the importance of following the specific manufacturer’s instructions for use. The panel highlighted the need for further research into optimal management strategies for these alternative devices, with the goal of reducing the risk of all HOUTIs.

**Related care interventions**

**Topics 9–10**

It was highlighted that the current literature to support the use of antiseptic solutions for catheter care in patients with indwelling urinary catheters is weak. While the potential benefit of using CHG to clean the urethral meatus has been highlighted as a tool to reduce CAUTIs in emerging literature, ^12,13^ some findings have, to date, been relatively weak. ^14^ In line with current CAUTI guidance, the panel emphasized the use of antiseptic solutions as an unresolved issue. ^8^ Identifying it as an important area for additional research. For the non-catheterized patient, the literature to support the use of CHG treatment is even more sparse, with the panel calling for research to identify best practices for these patients. In the absence of evidence-based guidance, across both catheterized and non-catheterized patients, the panel cited the Universal intensive care unit (ICU) Decolonization Protocol from the Agency for Healthcare Research and Quality (AHRQ), ^15^ which outlines steps for CHG bathing both for incontinence and for lines and tubes.

**Specimens and cultures**

**Topics 11–12**

There was extensive discussion surrounding the use of urinalysis with reflex to culture as a screening tool for symptomatic hospitalized patients with or without indwelling urinary catheters. Urinalysis is a complex topic and requires significant discussion and risk assessment. While no consensus was reached on this topic, the panel agreed that the high negative predictive value of urinalysis positions it as a powerful tool for ruling out infection, and in cases where a urinalysis yields a negative result, reflex cultures can be avoided. A positive urinalysis on the other hand, while it should trigger a reflex culture, should only result in antibiotic use if there is clinical correlation with culture results. Pyuria is common in ASB ^16^ and in older women, and symptoms remain essential to distinguish ASB from symptomatic HOUTI. There are, however, exceptions in which urinalysis may be unreliable and a culture is necessary, including neutropenia or in some immunocompromised patients. Many geriatric patients show an atypical presentation of disease, with altered mental status being the only clear symptom, and in these patients, a urinalysis could prevent the use of unnecessary urine cultures. The panel highlighted the importance of clinical judgement, aligning to national definitions, and periodic reviews of protocols to align with stewardship goals and to be considered under the proposed Centers for Disease Control and Prevention (CDC) HOB metric. ^17,18^

The panel considered the use of molecular diagnostic techniques such as PCR-based pathogen identification on urine. While speed was noted as a benefit, this technique was called into question due to low accuracy and a lack of standardization. Multiplex molecular panels for diagnosis of UTI lack clinical data supporting their use in routine clinical care, and also have the potential to exacerbate inappropriate antibiotic use. ^19^ Noting this, the panel called for studies evaluating the clinical utility of molecular diagnostic techniques in various patient populations and care settings.

**Outcome assessment**

**Topic 13**

The panel agreed with existing guidance ^8,9^ that establishing surveillance protocols for non-CAUTI is an indispensable first step in developing effective prevention strategies, but uncertainty remained regarding the most effective ways and channels through which to do so. Without routine tracking of non-CAUTI hospital-onset UTIs, neither the true burden nor the variation in incidence across facilities is known, making benchmarking, standardized infection ratios, and targeted prevention a difficult task. The panel recommended that a formal research agenda, starting with defining, validating and then deploying consistent non-CAUTI criteria will help lay the groundwork for meaningful prevention strategies and quality-improvement interventions.

**References**

1. Lean K, Nawaz RF, Jawad S, Vincent C. Reducing urinary tract infections in care homes by improving hydration. BMJ Open Quality. 2019;8(3): e000563.
2. Booth J, Agnew R. Evaluating a hydration intervention (DRInK Up) to prevent urinary tract infection in care home residents: A mixed methods exploratory study. Journal of frailty, sarcopenia and falls. 2019;4(2):36.
3. Gauhar V, Castellani D, Teoh JY, et al. Catheter-associated urinary infections and consequences of using coated versus non-coated urethral catheters—outcomes of a systematic review and meta-analysis of randomized trials. Journal of Clinical Medicine. 2022;11(15):4463.
4. Karchmer TB, Giannetta ET, Muto CA, et al. A randomized crossover study of silver-coated urinary catheters in hospitalized patients. Archives of Internal Medicine*.* 2000;160(21):3294–3298.
5. Rupp ME, Fitzgerald T, Marion N, et al. Effect of silver-coated urinary catheters: efficacy, cost-effectiveness, and antimicrobial resistance. American Journal of Infection Control. 2004;32(8):445–450.
6. Lederer JW, Jarvis WR, Thomas L, Ritter J. Multicenter cohort study to assess the impact of a silver-alloy and hydrogel-coated urinary catheter on symptomatic catheter-associated urinary tract infections. Journal of Wound Ostomy & Continence Nursing. 2014;41(5):473–480.
7. Kai-Larsen Y, Grass S, Mody B, et al. Foley catheter with noble metal alloy coating for preventing catheter-associated urinary tract infections: a large, multi-center clinical trial. Antimicrobial Resistance & Infection Control. 2021;10(1):40.
8. Patel PK, Advani SD, Kofman AD, et al. Strategies to prevent catheter-associated urinary tract infections in acute-care hospitals: 2022 Update. Infection Control & Hospital Epidemiology. 2023;44(8):1209–1231.
9. Crapanzano-Sigafoos R, Phillips B, Ormsby J, et al. Guide to Preventing Catheter-Associated Urinary Tract Infections (CAUTI). Arlington, VA: The Association for Professionals in Infection Control and Epidemiology (APIC). 2025.
10. Rosenthal VD, Memish ZA, Nicastri E, et al. Preventing catheter-associated urinary tract infections: a position paper of the International Society for Infectious Diseases, 2024 update. International Journal of Infectious Diseases. 2024:107304.
11. Hooton TM, Bradley SF, Cardenas DD, et al. Diagnosis, prevention, and treatment of catheter-associated urinary tract infection in adults: 2009 International Clinical Practice Guidelines from the Infectious Diseases Society of America. Clinical Infectious Diseases. 2010;50(5):625–663.
12. Mitchell, B, Curryer, C, Holliday, et al. Effectiveness of meatal cleaning in the prevention of catheter-associated urinary tract infections and bacteriuria: an updated systematic review and meta-analysis. BMJ Open. 2021;11:e046817.
13. Fasugba, O, Cheng, AC, Gregory, V, et al. Chlorhexidine for meatal cleaning in reducing catheter-associated urinary tract infections: a multicentre stepped-wedge randomised controlled trial. Lancet Infectious Diseases. 2019;19:611–619.
14. Huang SS, Septimus E, Kleinman K, et al. ABATE Infection trial team. Chlorhexidine versus routine bathing to prevent multidrug-resistant organisms and all-cause bloodstream infections in general medical and surgical units (ABATE Infection trial): a cluster-randomised trial. Lancet. 2019;393(10177):1205–1215.
15. Agency for Healthcare Research & Quality. Universal ICU Decolonization: An Enhance Protocol. Available at: <https://www.ahrq.gov/hai/universal-icu-decolonization/universal-icu-ape3.html> Last accessed April 2025.
16. Bilsen MP, Aantjes MJ, van Andel E, et al. Current pyuria cutoffs promote inappropriate urinary tract infection diagnosis in older women. Clinical Infectious Diseases. 2023 Jun 15;76(12):2070–2076.
17. Howard-Anderson J, Morgan DJ. Moving Beyond Central Line-Associated Bloodstream Infections. Annals of Internal Medicine. 2024;177(6):822–823.
18. Classen DC, Rhee C, Dantes RB, Benin AL. Healthcare-associated infections and conditions in the era of digital measurement. Infection Control & Hospital Epidemiology. 2024;45(1):3–8
19. Hatfield KM, Kabbani S, See I, et al. Use of multiplex molecular panels to diagnose urinary tract infection in older adults. JAMA Network Open. 2024;7(11):e2446842–.
