# Supplementary material for: Prevention Strategies for All Hospital-Onset Urinary Tract Infections: Best Practice Consensus Recommendations
Source: Open Forum Infect Dis. 2026 Feb 6;13(2):ofag060. doi: 10.1093/ofid/ofag060 (PMC12919440; doi:10.1093/ofid/ofag060)
Supplement: ofag060_Supplementary_Data [file ofag060_supplementary_data.zip › Supplementary material E_Areas of moderate consensus.docx]

**Areas of moderate consensus**

| **Intervention placement** |  |
| --- | --- |
| Indwelling urinary catheters may be considered for the following indications:   - Perioperative use following select surgical procedures - Management of acute urinary retention - When precise hourly assessment of bladder output is required - Assistance with healing certain open wounds or skin grafts - As part of end-of-life care regimen for pain or comfort management | 88%  88%  82%  82%  82% |
| **Intervention maintenance** |  |
| Waterless cleansing of the perineal area with a disposable incontinence cloth should be performed for bed bound patients with or without an indwelling urinary catheter | 76% |
| Perineal care with a waterless disposable cloth should be performed in female patients with external suction catheters. | 76% |
| To confirm catheterization is clinically indicated, bladder volume should be measured non-invasively with a bladder scanner prior to every intermittent straight catheterization, except in patients on an intermittent catheterization schedule | 82% |
| **Related care interventions** |  |
| Hydration protocols should be validated in acute care settings and implemented as part of hospital-onset UTI prevention strategies | 76% |
| Fecal management is necessary to mitigate the risk of hospital-onset UTI | 76% |
