# Supplementary material for: Prevention Strategies for All Hospital-Onset Urinary Tract Infections: Best Practice Consensus Recommendations
Source: Open Forum Infect Dis. 2026 Feb 6;13(2):ofag060. doi: 10.1093/ofid/ofag060 (PMC12919440; doi:10.1093/ofid/ofag060)
Supplement: ofag060_Supplementary_Data [file ofag060_supplementary_data.zip › Supplementary material B_HOUTI prevention_First survey_v3.docx]

**All Hospital-Onset UTI Prevention Delphi panel – First round survey questions**

**Scope of work:**The following questions and recommendations apply specifically to hospitalized adult patients, and are intended to be answered from the perspective of preventing both CAUTIs and non-CAUTIs. 

**Working definitions:**
Various definitions are provided in the literature depending on clinical contexts. For the purpose of this work, the following pragmatic definitions are proposed:

Urinary tract infections (UTI) are defined using the CDC Symptomatic Urinary Tract Infection (SUTI) criteria.

**Catheter-associated UTI (CAUTI):**

- **The patient must meet all three of the following:**

1. Patient had an indwelling urinary catheter that had been in place for more than 2 consecutive days in an inpatient location on the date of event AND was either:
   - Present for any portion of the calendar day on the date of event,

**OR**

- - Removed the day before the date of event

1. Patient has at least ***one*** of the following signs or symptoms:
   - fever (>38.0°C)
   - suprapubic tenderness with no other recognized cause
   - costovertebral angle pain or tenderness with no other recognized cause
   - urinary urgency ^
   - urinary frequency ^
   - dysuria ^
     1. *These symptoms cannot be used when an indwelling urinary catheter (IUC) is in place*
2. Patient has a urine culture with no more than two species of organisms identified, at least one of which is a bacterium of ≥105 CFU/ml.

**Non-catheter-associated UTI (non-CAUTI):**

- **The patient must meet all three of the following:**

1. One of the following is true:
   - Patient has/had an indwelling urinary catheter, but it has/had not been in place for more than two consecutive days in an inpatient location on the date of event

**OR**

- - Patient did not have an indwelling urinary catheter in place on the date of event nor the day before the date of event

1. Patient has at least ***one*** of the following signs or symptoms:
   - fever (>38°C)
   - suprapubic tenderness with no other recognized cause
   - costovertebral angle pain or tenderness with no other recognized cause
   - urinary frequency ^
   - urinary urgency ^
   - dysuria ^
     1. *These symptoms cannot be used when an indwelling urinary catheter (IUC) is in place*
2. Patient has a urine culture with no more than two species of organisms identified, at least one of which is a bacterium of ≥10^5^ CFU/ml.

**Pyuria**:

- A white blood cell count ≥10/mm^3^

| **Topic** | **Question number** | **Clinical question** | **Answer options** |
| --- | --- | --- | --- |
| *N/A* | *1–3* | *Screener questions – name, email, number* | *N/A* |
| Surveillance | 4 | It is important to define, monitor and report infection rates in units caring for patients with, or at risk of hospital-onset UTIs | True / false |
| Surveillance | 5 | Non-CAUTI hospital-onset UTI rates should be monitored and reported: [select only one] | - Daily - Weekly - Monthly - Quarterly - Other |
| Surveillance | 6 | Consistent diagnostic criteria and definitions (e.g., positive culture with clinical signs and symptoms) should be used across all units to identify both CAUTI and non-CAUTI hospital-onset UTIs | True / false |
| Surveillance | 7 | The use of electronic systems such as electronic health records (EHRs) is recommended over manual methods for infection surveillance and monitoring | True / false |
| Surveillance | 8 | EHRs should include urine management system documentation (e.g. indwelling urinary catheter, intermittent straight catheter, external catheter, bedpan/urinal, etc.) and decision support tools to prompt transitions to less invasive options | True / false |
| Surveillance | 9 | Rounding / huddles carried out by the care team are an effective way of ensuring periodic checks are completed regarding the continued need for the current urine management intervention | True / false |
| Surveillance | 10 | Identifying the root cause / pathogen responsible for the infection is important to implementing appropriate prevention strategies for all hospital-onset UTIs | True / false |
| Intervention selection | 11 | Urine output management should be viewed as a continuum of progressively less invasive devices with a goal of restoring patients to independent or assisted voiding | True / false |
| Intervention selection | 12 | Additional preventive interventions (e.g., bladder care protocols, hydration support) should be initiated in at-risk patients: [select only one] | - At admission - Within 24-hours of risk identification - Only when clinical symptoms develop - Other |
| Intervention selection | 13 | Urine management device selection should be tailored to patient-specific factors (e.g., condition, urine output needs, incontinence): [select only one] | - Always - Often - Rarely - Never - Other |
| Intervention selection | 14 | Indwelling urinary catheters may be considered for the following indications [select all that apply]: | - Perioperative use following select surgical procedures - When precise hourly assessment of urine output is required - Management of acute urinary retention - Assistance with healing certain open wounds or skin grafts - As part of a palliative care regimen for pain or comfort management |
| Intervention selection | 15 | Understanding different device types and characteristics (e.g., external catheters, intermittent catheters, Coude-tip catheters, antimicrobial-coated devices, IUC size/diameter, tip configuration) is important in addressing and meeting patient needs | True / false |
| Intervention selection | 16 | Bladder scanners should be routinely used to evaluate urinary retention before considering catheterization for suspected urinary retention | True / false |
| Intervention selection | 17 | To reduce insertion trauma and insertion attempts in male patients requiring indwelling urinary catheters, Coudé-tip indwelling urinary catheters should be employed first in patients aged: [select only one] | - 40 and above - 50 and above - 60 and above - 70 and above - 75 and above - Only if standard-tip catheterization is unsuccessful - Never - Other |
| Intervention selection | 18 | Antimicrobial catheters should be used in all patients | True / false |
| Intervention selection | 19 | If antimicrobial catheters are used, they should be implemented for patients anticipated to be catheterized for: | - Less than 7 days - 8–14 days - 15–21 days - 22 days or longer - Never - Other |
| Intervention selection | 20 | Which strategies should be prioritized to avoid unnecessary catheterization and minimization of infection risk? (select all that apply) | - Bladder scanners - Clinical decision support tools - Nurse-driven protocols for device transitions and discontinuation - Nurse-driven protocols with physician order - Hydration protocols - Nursing protocols for voiding trials - Other |
| Intervention placement | 21 | Poor hand hygiene negatively affects patient outcomes in patients with, or at risk of, hospital-onset UTIs | True / false |
| Intervention placement | 22 | Adherence to aseptic technique during the insertion and handling of any devices should be mandatory to reduce UTIs | True / false |
| Intervention placement | 23 | Prior to the insertion of all intraurethral urine management interventions (e.g. indwelling urinary catheters and intermittent catheters), antiseptic solutions should be used for cleaning the urethral meatus | True / false |
| Intervention placement | 24 | The antiseptic solution should ideally be: | - Povidone iodine - Low-concentration CHG solution - Other - None |
| Intervention placement | 25 | Appropriate hygiene and a two-person / ‘four eyes’ approach during insertion /application should be standardized to reduce infection risk | True / false |
| Intervention placement | 26 | Intervention selection (e.g., selecting a closed-system indwelling urinary catheterization tray with a drainage bag versus a urine meter) at placement should be guided by patient-specific urine output monitoring needs and should be done at time of indwelling urinary catheter placement | True / false |
| Intervention placement | 27 | Sealed, pre-connected closed systems are recommended for urine output monitoring for appropriate infection control | True / false |
| Intervention placement | 28 | Catheter securement to prevent catheter movement should be employed in all patients with indwelling urinary catheters | True / false |
| Intervention placement | 29 | In patients who are catheterized, it is important to be aware of the hospital location in which the device was inserted (i.e., ED / OR / ICU / ward) | True / false |
| Intervention maintenance | 30 | Patients’ urine management systems should be reassessed to transition patients to less invasive approaches: [select all that apply] | - Every HCP shift - Twice daily - Daily - Weekly - Only when clinically indicated - Other |
| Intervention maintenance | 31 | A sealed, pre-connected closed drainage system should be maintained for all patients with indwelling urinary catheters to reduce infection risk | True / false |
| Intervention maintenance | 32 | Gravity-fed (non-suction-assisted) urine collection systems placed below the bladder level are critical to infection prevention | True / false |
| Intervention maintenance | 33 | Hygiene and inspection of patients with indwelling urinary catheters should take place: [select only one] | - Hourly - Every 4 hours - Every 8 hours - Every HCP shift - Daily - Other |
| Intervention maintenance | 34 | Meatal cleaning with non-alcohol antiseptic should be performed in patients with indwelling urinary catheters: [select only one] | - Hourly - Every 4 hours - Every 8 hours - Every HCP shift - Daily - Other |
| Intervention maintenance | 35 | Hygiene and inspection (including skin assessment) of patients with external urinary catheters (e.g. female and male wicking-type catheters, condom catheters) should take place: [select only one] | - Hourly - Every 4 hours - Every 8 hours - Every HCP shift - Daily - Other |
| Intervention maintenance | 36 | Meatal cleaning with non-alcohol antiseptic should be performed in patients with female external wicking-type catheters: [select only one] | - Hourly - Every 4 hours - Every 8 hours - Every HCP shift - Daily - Other |
| Intervention maintenance | 37 | Assessing patient-specific characteristics (e.g., treatment needs, injury, physical characteristics) is key to understanding the appropriate location to place a urine management system | True / false |
| Intervention maintenance | 38 | Indwelling urinary catheters, and their associated collection systems should be replaced (select all that apply): | - If the closed system is broken or compromised - If a urine specimen is required - If infection is diagnosed - If the catheter becomes blocked or fails to drain - Every 7 days - Every 14 days - Every 21 days - Other |
| Intervention maintenance | 39 | Female external catheters should be replaced: [select all that apply] | - Every 8 hours - Every 12 hours - Every HCP shift - Every 24 hours - When soiled or otherwise indicated - Other |
| Intervention maintenance | 40 | Male external wicking-type catheters should be replaced: [select all that apply] | - Every 8 hours - Every 12 hours - Every HCP shift - Every 24 hours - When soiled or otherwise indicated - Other |
| Intervention maintenance | 41 | Male external condom catheters should be replaced: [select all that apply] | - Every 8 hours - Every 12 hours - Every HCP shift - Every 24 hours - When soiled or otherwise indicated - Other |
| Intervention maintenance | 42 | In order to minimize unnecessary catheterizations, bladder volume should be measured non-invasively with a bladder scanner prior to every intermittent straight catheterization, to confirm that bladder volume is sufficient for catheterization. | True / false |
| Related care interventions | 43 | Hand hygiene protocols should be strictly followed before and after any interaction with a patient’s urinary system or device | True / false |
| Related care interventions | 44 | Hydration protocols should be implemented as part of hospital-onset UTI prevention strategies: [select all that apply] | - Always - Often - Rarely - Dependent on patient needs - Other |
| Related care interventions | 45 | CHG bathing to reduce the risk of all hospital-onset UTIs in high-risk patients should be performed: [select all that apply] | - Daily - Every other day - After an episode of fecal incontinence - Never - Other |
| Related care interventions | 46 | Targeted decolonization (e.g., skin antiseptics) plays a key role in reducing UTI risk | True / false |
| Related care interventions | 47 | Fecal management and containment is critical to mitigating the risk of hospital-onset infection | True / false |
| Specimens and cultures | 48 | Culture stewardship is a key component of high-quality infection control and patient care | True / false |
| Specimens and cultures | 49 | The use of clean catch, aseptic or sterile techniques in obtaining uncontaminated urine specimens is a key component of quality infection control | True / false |
| Specimens and cultures | 50 | Reflex urine cultures based on urinalysis results, such as pyuria, in concert with other symptoms (e.g. fever, flank pain, pelvic discomfort, altered mental status with symptoms) are appropriate | True / false |
| Specimens and cultures | 51 | In patients with an indwelling catheter, if a fresh urine specimen is required for culture, the catheter should be replaced if it has been in place for longer than: | - 3 days - 7 days - 14 days - 21 days - Other |
| Specimens and cultures | 52 | The adoption of molecular diagnostic techniques (e.g., PCR-based pathogen identification) is important in improving the speed and accuracy of UTI diagnosis in hospitalized patients | True / false |
| Provider training | 53 | Roles and responsibilities for urine management should be clearly defined across interdisciplinary teams | True / false |
| Provider training | 54 | Provider training should include device selection, placement, assessment/management, and removal | True / false |
| Provider training | 55 | Provider training should extend to individuals who may need to manipulate urine management systems, including aids and transport personnel | True / false |
| Provider training | 56 | Provider training should emphasize transitioning to less invasive urine management systems | True / false |
| Provider training | 57 | Provider training with competency assessment should be completed: [select only one] | - Every 6 months - Annually - Every 2 years - Other |
| Provider training | 58 | Interdisciplinary training (involving nursing, physicians, urology, infection control, etc.) on all hospital-onset UTI prevention and mitigation of all urine management trauma (e.g. insertion trauma, skin damage, etc.) should be conducted: [select only one] | - Every 6 months - Annually - Every 2 years - Other |
| Outcome assessment | 59 | In addition to setting definitions for CAUTIs, organizations should establish separate definitions for non-CAUTI hospital-onset UTIs | True / false |
| Outcome assessment | 60 | Infection rates for both CAUTI and non-CAUTI hospital-onset UTIs should be a key performance indicator for unit / departments managing patients at risk of hospital-onset UTIs | True / false |
| Outcome assessment | 61 | Granular utilization metrics to include indwelling urinary catheter-days, external catheter-days, intermittent straight catheterization days etc., should be collected to better understand device utilization | True / false |
| Outcome assessment | 62 | The quality of hospital-onset UTI management should be prospectively audited with metrics such as length of stay, antimicrobial use, device-related trauma, regression to more invasive devices, etc. | True / false |
